# Case Report: From septic shock to recovery – a battle against lethal necrotizing soft tissue infection

**DOI:** 10.3389/fimmu.2025.1680818

**Published:** 2025-09-26

**Authors:** Yimin Xue, Na Wang, Chaoyu Wu, Hongmei Yang, Xiongfei Zou, Huaiwu He, Yan Shi

**Affiliations:** ^1^ Department of Intensive Care Unit, Peking Union Medical College Hospital, Peking Union Medical College and Chinese Academy of Medical Sciences, Beijing, China; ^2^ Shengli Clinical Medical College of Fujian Medical University, Department of Cardiovascular Critical Care Medicine, Fujian Provincial Hospital, Fuzhou University Affiliated Provincial Hospital, Fuzhou, Fujian, China; ^3^ Department of Emergency Medicine, Guizhou Provincial People’s Hospital, Guiyang, Guizhou, China; ^4^ Department of Intensive Care Unit, Lijiang People’s Hospital, Lijiang, Yunnan, China; ^5^ Department of Orthopedic Surgery, Peking Union Medical College Hospital, Peking Union Medical College and Chinese Academy of Medical Sciences, Beijing, China

**Keywords:** necrotizing soft tissue infections, streptococcus pyogenes, septic shock, V-A ECMO, surgical debridement, multidisciplinary care

## Abstract

Necrotizing soft tissue infections (NSTIs) are life-threatening surgical emergencies associated with high morbidity and mortality. We report the case of a 40-year-old woman who presented with progressive left lower limb pain and swelling. CT revealed inflammatory changes involving the deep soft tissues. Despite initial surgical debridement, irrigation, and negative pressure wound therapy (NPWT), the patient rapidly deteriorated and progressed to septic shock, requiring intensive care unit (ICU) admission, broad-spectrum antibiotics, advanced life-sustaining support with veno-arterial extracorporeal membrane oxygenation (V-A ECMO), and continuous renal replacement therapy (CRRT). Cultures from the wound drainage identified *Streptococcus pyogenes* (*S. pyogenes*), leading to targeted antimicrobial therapy. Ongoing clinical deterioration and elevated myoglobin levels prompted repeated surgical debridements for progressive muscle and skin necrosis. Following multiple staged debridements and multidisciplinary management, the patient achieved hemodynamic stability, successful wound closure, and skin grafting. This case highlights the critical need for timely diagnosis, aggressive surgical and medical management, and coordinated multidisciplinary care in improving outcomes for patients with severe NSTIs.

## Introduction

Necrotizing soft tissue infection (NSTI) is a severe and potentially fatal infection that leads to progressive necrosis of the skin, subcutaneous tissue, fascia, and underlying muscle tissue (1, 2). It typically presents with rapid clinical deterioration, extensive soft tissue destruction, and a high mortality rate, particularly when diagnosis and treatment are delayed (2). NSTIs can be caused by a variety of pathogens, including bacteria such as *group A Streptococcus* (GAS), *Staphylococcus aureus*, *Clostridium perfringens*, and *mixed aerobic-anaerobic organisms* (3). The infection often originates from minor trauma, surgical wounds, or hematogenous spread, and it may rapidly escalate due to the release of bacterial toxins that induce a hyperinflammatory response and impair local perfusion (4). Early recognition, aggressive surgical debridement, broad-spectrum antimicrobial therapy, and intensive supportive care are critical for improving outcomes (5, 6). Despite advances in medical and surgical management, NSTI remains a life-threatening clinical emergency requiring a multidisciplinary approach. Herein, we report the successful management of a severe case of NSTI, aiming to provide insights into its clinical approach and enhance diagnostic and therapeutic strategies.

## Case presentation

**Figure 1:**
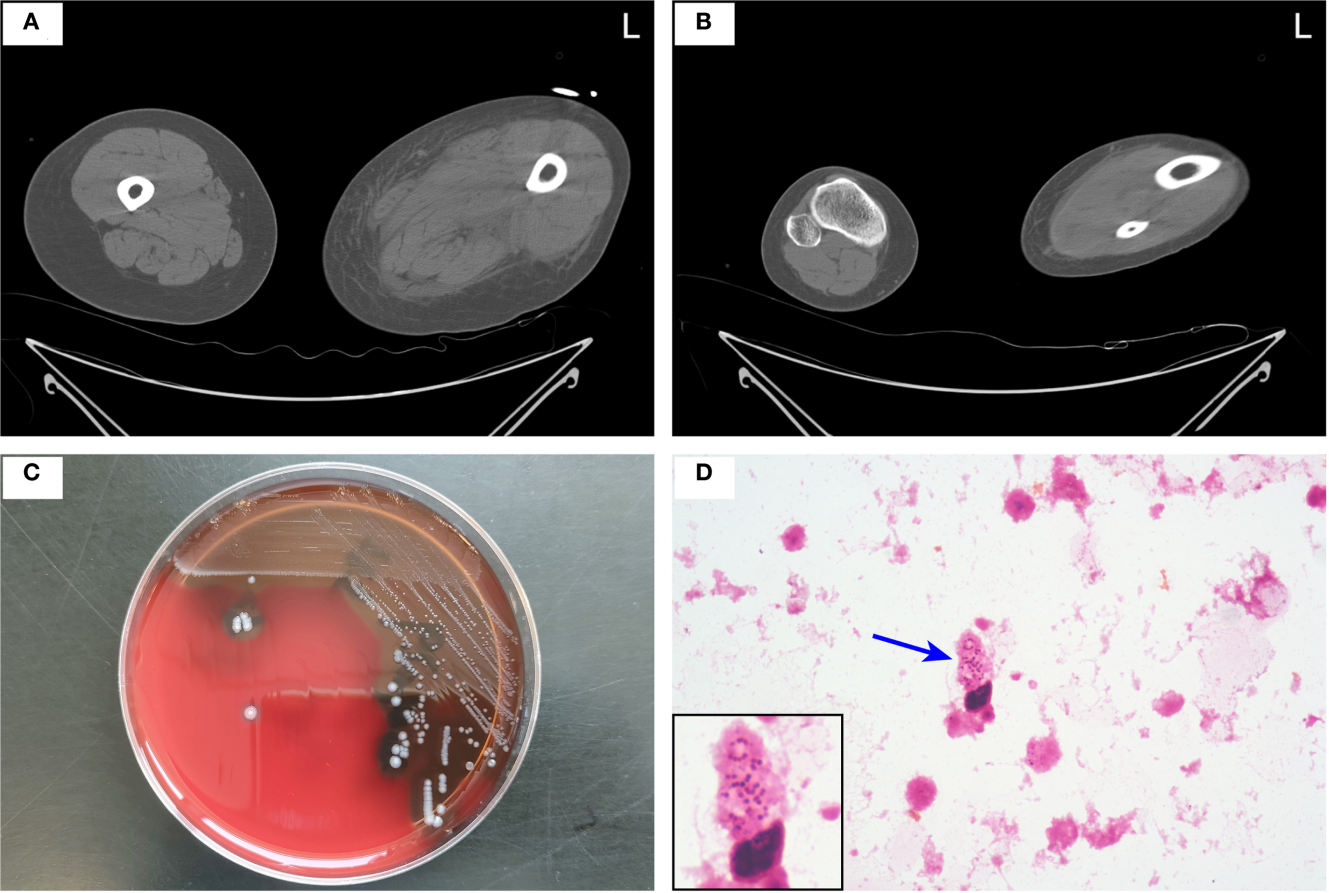
**(A, B)** Axial CT scan of both lower limbs showing inflammatory changes in the intramuscular spaces and subcutaneous fat of the medial posterior aspect of the left thigh **(A)** and lower leg **(B)**. **(C, D)** Drainage fluid was aseptically inoculated into aerobic and anaerobic BACTEC™ culture bottles (BD, Franklin Lakes, NJ, USA) and incubated at 37°C for 24 hours. Upon instrument-determined positivity, the broth was subcultured onto a 5% sheep blood agar plate and incubated at 37°C in 5% CO_2_ for 24 hours, yielding small, beta-hemolytic colonies **(C)**. Gram staining of colony material revealed Gram-positive cocci in chains at 1000× magnification, with the chain indicated by a blue arrow **(D)**. *S. pyogenes* was confirmed by catalase negativity and Lancefield group A antigen detection.

**Figure 2:**
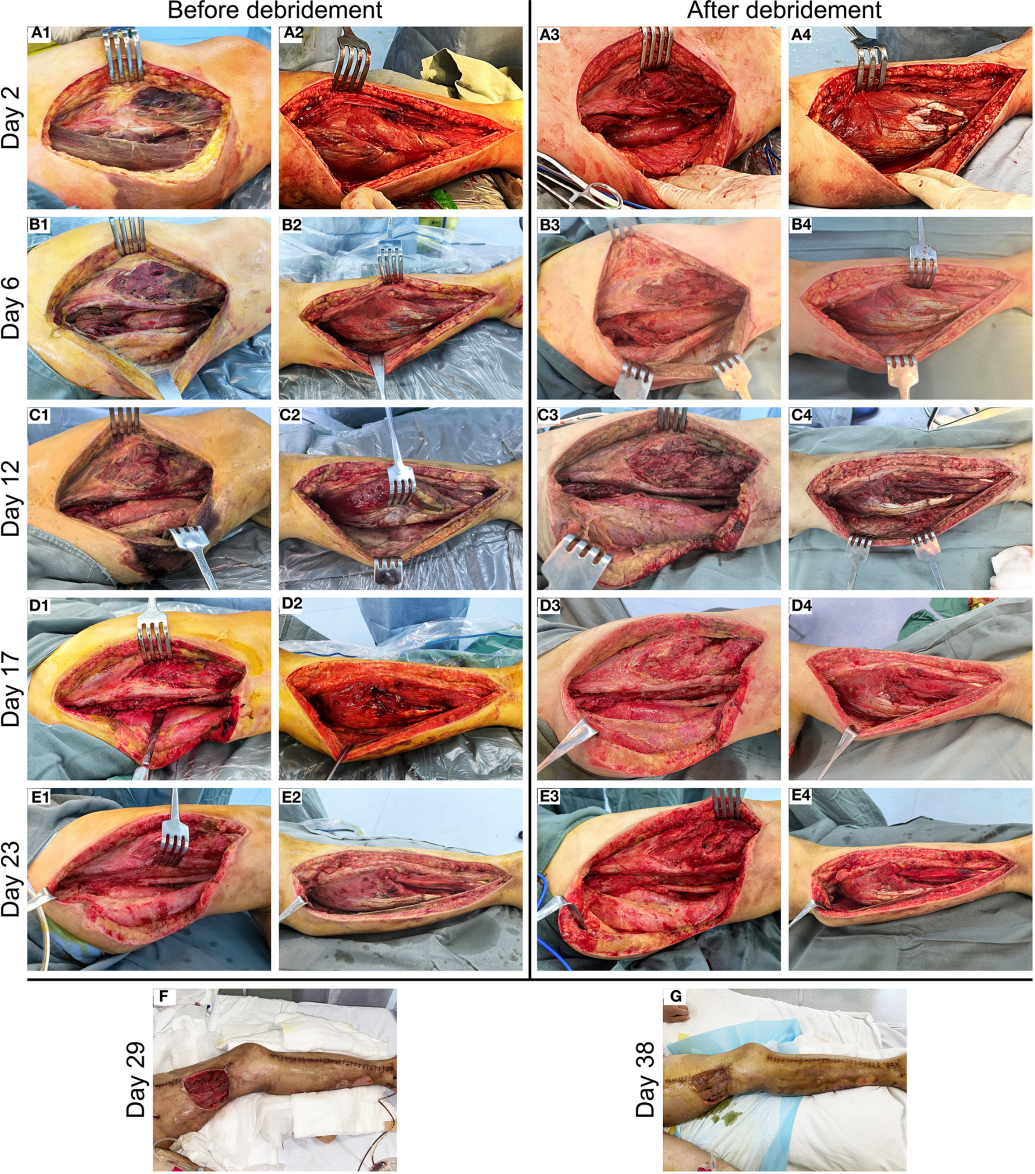
Clinical progression of wound healing in the patient with NSTI of the left lower limb. **(A)** Necrotic lesions detected in the medial thigh musculature **(A1)** and gastrocnemius muscle **(A2)** on postoperative day 2, followed by complete debridement of nonviable tissue **(A3, A4)**. **(B–E)** Preoperative and postoperative photographs of staged serial debridement of the left lower extremity, performed on day 6 **(B1–B4)**, day 12 **(C1–C4)**, day 17 **(D1-D4)**, and day 23 **(E1–E4)**, respectively. **(F)** Postoperative view of the wound with partial suturing performed on day 29. **(G)** Appearance of the left thigh flap transplant site on day 38.

A 40-year-old woman presented to the emergency department with a 5-day history of left lower extremity pain and swelling that had worsened acutely, accompanied by persistent fever with a recorded maximum temperature of 40°C. Upon arrival in the resuscitation bay, the patient was lethargic with clammy skin and exhibited fever (38.5°C), tachycardia (120 bpm), hypotension (85/44 mmHg) on norepinephrine (0.5 μg/kg/min), tachypnea (24 breaths/min), and SpO_2_ 97% on 3 L/min oxygen via nasal cannula. She had previously been diagnosed with left knee joint synovitis, but denied any recent treatment or trauma. The CT scan revealed mild thickening of the soft tissues in the medial posterior region of the left lower limb, accompanied by inflammatory changes in the intramuscular spaces and subcutaneous fat (**Figures 1A, B**). After informed consent was obtained, the patient underwent surgical incision of the left thigh, drainage, and irrigation under general anesthesia. During the operation, approximately 500 ml of grayish-red fluid was flushed from the tissue spaces, and no tissue necrosis was observed. Due to profound hemodynamic instability and rapid progression to septic shock (**Supplementary Video 1A**), the patient was transferred to the intensive care unit (ICU) on an emergency basis following the application of negative pressure wound therapy (NPWT), and was promptly initiated on empiric broad-spectrum antibiotic therapy (meropenem 1g IV q6h combined with linezolid 600mg IV q12h) and supported with veno-arterial extracorporeal membrane oxygenation (V-A ECMO) and continuous renal replacement therapy (CRRT). On the first postoperative day, *Streptococcus pyogenes* (*S. pyogenes*) was identified in the drainage fluid sample (**Figures 1C, D**), prompting a change in antibiotic therapy to amoxicillin-clavulanate 2.4g IV q8h and clindamycin 900mg IV q6h. Although the patient’s hemodynamics stabilized gradually, she developed noticeable swelling and ecchymosis around the surgical site by postoperative day 2. Laboratory findings revealed persistently elevated myoglobin (Myo) levels exceeding 100,000 ng/mL and sustained lactate concentrations of approximately 5.0 mmol/L, along with high levels of inflammatory markers, including white blood cell count (WBC, 30.23 × 10^9^/L), C-reactive protein (CRP > 304.00 mg/L), and procalcitonin (PCT, 43.22 ng/mL), all of which were strongly indicative of extensive muscle necrosis. Accordingly, a second surgical debridement was performed on postoperative day 2 (**Figure 2A**), during which necrotic changes were confirmed in the medial thigh muscles and gastrocnemius of the lower leg. Most of the nonviable muscle tissue was debrided. Subsequently, the patient achieved hemodynamic stability, with marked improvement in cardiac function (**Supplementary Video 1B**), and was successfully weaned off V-A ECMO on day 4 without complications.

**Figure 3:**
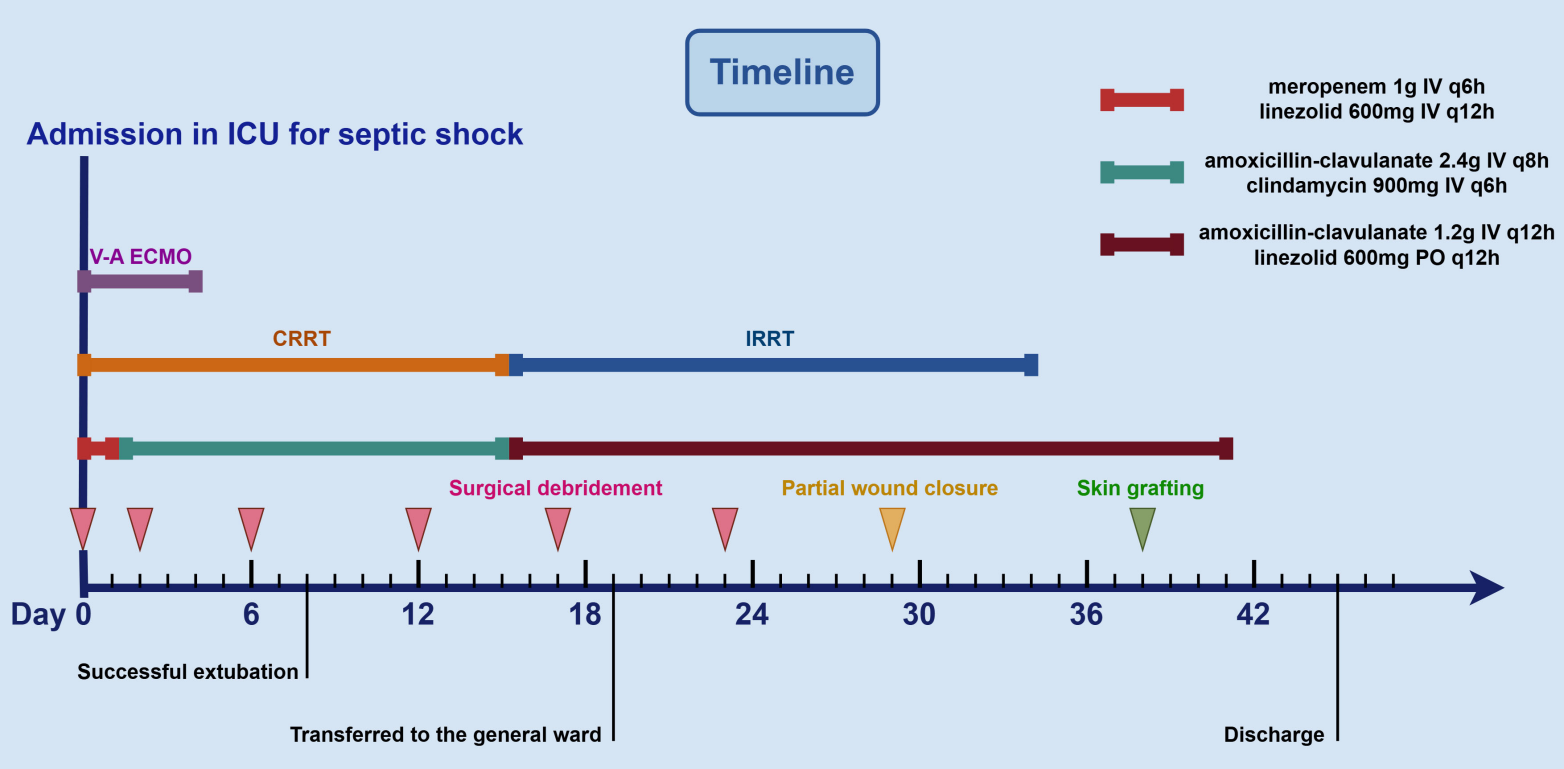
The timeline and treatment course for the patient.

On day 6, the patient showed a gradual decline in both inflammatory markers (WBC count decreased to 15.14 × 10^9^/L, CRP decreased to 82.45 mg/L, and PCT decreased to 3.56 ng/mL) and serum Myo levels (3,839 ng/mL), and no pathogens were isolated from the drainage culture. However, intermittent low-grade fever persisted, with body temperature fluctuating between 37.3°C and 37.8°C. Therefore, debridement was repeated to remove residual necrotic muscle tissue (**Figure 2B**), and NPWT was continued. On day 8, the patient regained full consciousness after discontinuation of sedation, exhibited effective spontaneous coughing, and maintained adequate oxygenation, leading to successful extubation, and early rehabilitation was initiated. Despite improvement in clinical symptoms, progressive blackening and necrosis of the skin at the medial aspect of the thigh incision were observed. Laboratory monitoring revealed a subsequent rise in inflammatory markers (WBC count increased to 22.68 × 10^9^/L, CRP increased to 132.34 mg/L, and PCT increased to 8.77 ng/mL) compared to previous levels. On day 12, an extended debridement was performed to debride necrotic skin and subcutaneous tissue from the medial thigh, as well as involved portions of the soleus muscle in the lower leg (**Figure 2C**). The patient’s condition stabilized progressively, with resolution of fever. RRT was successfully transitioned to an intermittent regimen (IRRT) on day 15. On day 17, planned surgical debridement was performed to address minimal residual necrotic tissue, involving sharp excision and thorough irrigation with saline and antiseptic solutions (**Figure 2D**). The patient was transferred to a general ward on day 19, where rehabilitation therapy was continued. A final debridement on day 23 confirmed complete clearance of nonviable tissue, with copious irrigation to optimize the wound bed in preparation for closure (**Figure 2E**). Partial wound closure was achieved on day 29 (**Figure 2F**), followed by definitive skin grafting on day 38 (**Figure 2G**). Due to clindamycin intolerance manifesting as diarrhea, the antibiotic regimen was changed on day 15 to intravenous amoxicillin-clavulanate 1.2 g q12h combined with oral linezolid 600 mg q12h, and antibiotics were ultimately discontinued on day 41. Fortunately, spontaneous urine output returned on day 34, allowing successful withdrawal of IRRT. The patient was eventually discharged on day 45 (**Figure 3**). At the two-week follow-up, the patient continued to exhibit deficits in hip adduction and abduction, as well as ankle plantar flexion, with no signs of systemic bacterial infection.

## Discussion

This case details the successful management of a 40-year-old woman with NSTI caused by *S. pyogenes*, complicated by severe septic shock requiring V-A ECMO and CRRT. Despite the life-threatening nature of the infection, a favorable outcome was achieved through timely diagnosis, aggressive surgical intervention, and comprehensive multidisciplinary critical care support. This case underscores the critical importance of early recognition, rapid escalation of life-sustaining therapies, and serial debridement in controlling the infection and preserving limb function.

NSTIs are rare but devastatingly aggressive and life-threatening conditions characterized by rapid progression and extensive tissue necrosis, often leading to septic shock and multi-organ failure if not promptly treated. These infections may arise from minor or major trauma in otherwise healthy individuals, from chronic wounds in immunocompromised patients, or following intramuscular injections of nonsteroidal anti-inflammatory drugs (7). Notably, approximately one-quarter of cases lack an identifiable portal of entry, posing a significant diagnostic challenge (7, 8). As illustrated in this case, the absence of a clear entry point may contribute to a missed or delayed diagnosis, allowing unchecked disease progression and resulting in severe clinical deterioration. Early recognition is crucial and depends on identifying key clinical “red flags”, including severe pain that is disproportionate to physical findings, rapidly spreading erythema and edema, skin discoloration (e.g., purple or bronze hue), hemorrhagic bullae, crepitus, and focal anesthesia due to nerve involvement (9, 10). These local manifestations are frequently accompanied by systemic toxicity, such as fever, tachycardia, hypotension, or altered mental status, further heightening clinical suspicion even in the absence of definitive imaging or laboratory results (11, 12). In this case, pain out of proportion to clinical examination, accompanied by progressive swelling and early signs of systemic instability, prompted immediate surgical exploration. While definitive debridement of all necrotic tissue was not achieved during the initial intervention, prompt surgical incision, drainage, and extensive irrigation played a critical role in managing the infection. This approach effectively reduced the local bacterial load and facilitated the clearance of toxins and inflammatory debris. By establishing adequate drainage, compartment pressure was relieved, microcirculation improved, and the spread of infection was contained. Furthermore, these measures helped mitigate the systemic inflammatory response and created favorable local conditions for subsequent, more definitive debridement procedures. Indeed, the timing of initial surgical intervention remains one of the few modifiable prognostic factors in NSTIs, with improved outcomes consistently observed in patients undergoing source control within hours of admission compared to those with delays exceeding 12 hours (5, 13, 14). Of course, dynamic assessment following initial debridement is also crucial, since the extent of tissue involvement may progress, as seen in our patient. Therefore, timely source control through incision, drainage, and irrigation is a vital first step in the management of severe NSTIs, even when complete necrosectomy is deferred, laying the groundwork for successful staged surgical management.

In this case, the patient rapidly progressed to septic shock with worsening organ dysfunction, necessitating immediate initiation of V-A ECMO and CRRT. This advanced support rapidly restored hemodynamic stability, which is a prerequisite for subsequent aggressive debridement, allowing the patient to tolerate extensive surgical procedures and minimizing perioperative risks. A series of surgical debridements (performed on postoperative days 2, 6, 12, 17, and 23) was conducted based on clinical findings and serial inflammatory marker trends, allowing for thorough excision of necrotic tissue while sparing viable muscle and skin. The decision to pursue serial debridement rather than early amputation was made in consideration of preserved neurovascular integrity and the absence of diffuse ischemia, reflecting a limb-salvage strategy consistent with current evidence-based practices (15, 16). Studies have demonstrated that, in the absence of irreversible limb ischemia, serial debridement is associated with survival rates comparable to those achieved with amputation, while offering significantly improved functional outcomes and quality of life (6, 17). This limb-salvage paradigm is further supported by clinical outcomes across multiple cohorts. For instance, Crowe et al. reported over 90% survival with limb preservation in cases of upper extremity NSTIs, and more recent data, including those from Park et al., demonstrate that multidisciplinary, stepwise surgical management enables limb salvage in nearly 80% of cases without compromising mortality (18, 19). Hypotension at admission, admission glucose > 300 mg/dL, and a LRINEC score > 9 were identified as independent predictors of limb loss (19). Notably, NPWT was initiated during the inter-debridement phase to facilitate wound bed preparation and reduce exudate. However, in retrospect, the early application of NPWT may have posed a risk by potentially concealing ongoing tissue necrosis. An open wound management strategy following initial debridement allows for direct assessment of tissue progression and may enable earlier intervention, regardless of laboratory markers or serum myoglobin levels. Therefore, while NPWT offers valuable benefits in promoting granulation tissue formation and wound stabilization, it should ideally be deferred until surgical confirmation of adequate source control has been achieved (20, 21). This consideration serves as an important reminder for surgeons managing similar cases in the future. These comprehensive interventions ultimately established effective source control, paving the way for classification-directed antimicrobial therapy.

NSTIs are typically classified into two primary types based on microbial etiology: Type I, which is polymicrobial and often involves both aerobic and anaerobic bacteria, typically seen in patients with underlying conditions such as diabetes or peripheral vascular disease; and Type II, which is monomicrobial, predominantly caused by *S. pyogenes* (GAS), frequently affecting otherwise healthy individuals and characterized by rapid progression and systemic toxicity (1, 22). However, additional subtypes exist, including Type III infections, primarily associated with marine pathogens like *Vibrio vulnificus*, and Fournier’s gangrene, a form affecting the perineal region (3). Our patient’s clinical presentation and positive cultures for *S. pyogenes* were consistent with Type II NSTI, necessitating targeted antimicrobial therapy. Although penicillin-based regimens remain first-line for GAS infections, we selected amoxicillin-clavulanate in combination with clindamycin to enhance both bactericidal efficacy and toxin suppression. Amoxicillin-clavulanate provides reliable killing of *S. pyogenes*, while clindamycin inhibits bacterial protein synthesis, thereby reducing the production of streptococcal pyrogenic exotoxins and attenuating the systemic inflammatory response that drives capillary leak and organ dysfunction (23). Notably, Heath et al. reported that the use of clindamycin in the initial antibiotic regimen for NSTI treatment was shown to significantly decrease rates of amputation—a finding that underscores its clinical impact beyond antimicrobial activity (24). This immunomodulatory effect has been associated with improved outcomes in streptococcal toxic shock syndrome and is now considered a cornerstone of severe GAS infection management (25). However, the patient developed clindamycin-induced diarrhea during treatment, necessitating discontinuation. Linezolid was substituted to preserve protein synthesis inhibition, suppress toxin production, and ensure effective anti-streptococcal coverage with reliable tissue penetration (23, 26). The combination of amoxicillin-clavulanate and linezolid was continued until clinical stability, successful skin grafting, and resolution of inflammatory markers. Given the extent of tissue loss and the need for delayed reconstruction, antibiotic therapy was extended to ensure infection control, although current guidelines recommend shorter durations following adequate surgical debridement.

The patient continues to attend regular follow-up appointments at the plastic surgery and rehabilitation outpatient clinics, demonstrating steady functional improvement. Rehabilitation efforts will focus on maximizing remaining muscle function and implementing compensatory strategies to enhance mobility. This case underscores several critical lessons. First, maintaining a high level of suspicion for NSTIs is essential—even in the absence of classic risk factors. Second, survival and limb salvage depend on early diagnosis, prompt surgical debridement, and aggressive multidisciplinary supportive care, including life-sustaining therapies such as V-A ECMO and CRRT when indicated. Third, repeated surgical evaluations are crucial due to the rapid progression of tissue necrosis. Definitive source control should be achieved through earlier and more extensive initial debridement, with NPWT deferred until infection is confidently ruled out. Fourth, antimicrobial management requires combining broad-spectrum initial coverage with a timely transition to pathogen-directed therapy, incorporating clindamycin for its toxin-suppressive properties. Ultimately, successful outcomes rely on seamless coordination among surgeons, intensivists, infectious disease specialists, and rehabilitation professionals—a truly multidisciplinary approach is integral to optimal care for these critically ill patients.

## Conclusion

We report a case of severe NSTI in an immunocompetent patient who achieved survival and meaningful functional recovery following early multimodal intervention. The integration of advanced organ support, staged surgical debridement, precise antimicrobial therapy, and early rehabilitation highlights the necessity of a coordinated, multidisciplinary approach. Importantly, the patient’s reported satisfaction with her recovery emphasizes that successful outcomes in NSTIs must encompass not only survival, but also long-term quality of life and functional well-being.

## Data Availability

The original contributions presented in the study are included in the article/**Supplementary Material**. Further inquiries can be directed to the corresponding author.
