# Supplementary material for: Case Report: From septic shock to recovery – a battle against lethal necrotizing soft tissue infection
Source: Front Immunol. 2025 Sep 26;16:1680818. doi: 10.3389/fimmu.2025.1680818 (PMC12511119; doi:10.3389/fimmu.2025.1680818)
Supplement: Supplementary file 1 [file Presentation1.pptx]

## Slide 1
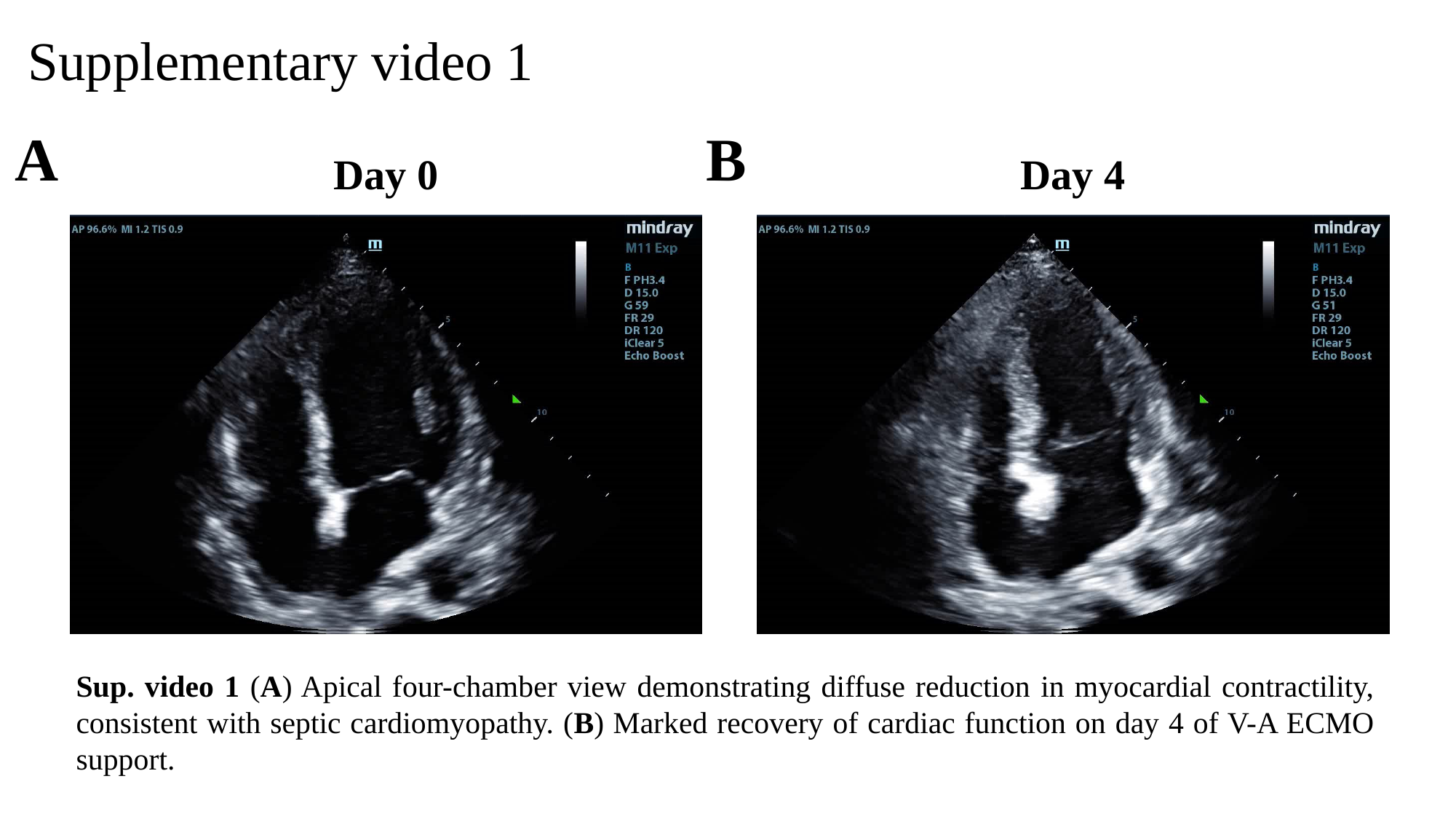

Supplementary video 1
B
A
Day 0
Day 4
Day 1
Day 1
Sup. video 1 (A) Apical four-chamber view demonstrating diffuse reduction in myocardial contractility, consistent with septic cardiomyopathy. (B) Marked recovery of cardiac function on day 4 of V-A ECMO support.
